# Correlation between gene mutation status and clinicopathologic features in early multiple primary lung cancer

**DOI:** 10.3389/fonc.2023.1110259

**Published:** 2023-04-12

**Authors:** Fei Teng, Jian Xu, Jian Wang, Bo Yang, Yong-Zhong Wu, Yue-Quan Jiang, Zhi-Qiang Wang

**Affiliations:** ^1^ Key Laboratory for Biorheological Science and Technology of Ministry of Education (Chongqing University), Chongqing University Cancer Hospital, Chongqing, China; ^2^ Department of Neurovascular Surgery, The First Hospital of Jilin University, Changchun, China; ^3^ Department of Thoracic Surgery, The First Hospital of Jilin University, Changchun, China

**Keywords:** clinicopathological features, demographic characteristics, gene mutation, ground-glass nodule, multiple primary lung cancer

## Abstract

**Objective:**

To understand the characteristics of genetic mutation in multiple primary lung cancer so as to guide clinical decisions in targeted therapy.

**Methods:**

We analyzed a total of 265 tumors from 111 patients who underwent surgery for multiple lung cancers. Individual tumors were subjected to histological evaluation and gene mutation analysis using ABI 7500 Fluorescence quantitative PCR.

**Results:**

In this study, we analyzed demographic and clinical parameters such as age, gender, smoking, alcohol consumption, pathological type, number of nodules, and other details of 111 patients with early multiple primary lung cancer. We also compared the clinicopathologic characteristics of different populations based on the gene mutation status of pulmonary nodules. Subsequently, we performed a clinicopathological analysis of all 265 pulmonary nodules from these patients. Results showed significant differences in clinicopathological features of pulmonary nodules in different genetic mutations.

**Conclusion:**

This study revealed the gene mutation characteristics and clinicopathological features in early multiple primary lung cancer. We found that the gene mutation status between different nodules in patients with early multiple primary lung cancer was inconsistent in most cases. Therefore, the use of targeted therapy based on the genetic sequencing of only one nodule, is unreliable. We hope this study can be helpful in guiding clinical treatment decisions.

## 1 Introduction

Lung cancer ranks second in the morbidity of malignant tumors worldwide, accounting for 11.4% of novel cancers (1). It is the leading cause of cancer deaths, with global mortality of 1.8 million per year (1). In particular, the five-year survival rate is only 10%–20% among patients with middle or advanced stages of cancer. With the popularization of thin-section CT and the increase in the number of patients screened for respiratory diseases during the COVID-19 pandemic, the detection rate of early-stage lung cancer has increased significantly (2, 3). Surgical treatment is mainly used for early-stage lung cancer, with a five-year survival rate of up to 70%–90% (4). The five-year survival rate can even approach 100%, especially for lung cancer with radiographic manifestations of ground-glass nodules. It is therefore possible to improve the overall survival rate of lung cancer *via* early screening and surgical treatment. However, there are primary malignant cases characterized by multiple nodules including multiple ground-glass nodules in patients with pulmonary nodules in early-stage lung cancer, which is termed multiple primary lung cancer. Multiple primary lung cancer refers to two or more primary cancers in different sites of one or both lungs, with either consistent or different histology but with no association between the two cancers. Based on the time when the tumors are identified, the disease can be classified as synchronous or metachronous. Multiple primary lung cancers have been reported to account for 0.7%–15% of lung cancers (5–9). Similar to single lung cancer, early multiple primary lung cancer in particular, is still mainly treated by surgical resection. However, total resection with surgery may not be feasible in some patients due to scattered lesions, poor pulmonary function, postoperative recurrence, refusal of multiple operations, and so on, posing challenges in the treatment of multiple primary lung cancer. There is no reference for the treatment of residual pulmonary nodules and use of targeted drugs.

With rapid advances in modern molecular biology technology, the treatment model for lung cancer is focused on targeting abnormal molecules in specific signaling pathways (10, 11). In the past decade, molecular targeted therapies represented by tyrosine kinase inhibitors (TKIs) have demonstrated significant clinical efficacy, paving the way for effective treatment of lung cancer (12, 13). The gene mutation rate of patients with lung cancer presenting EGFR as the main genotype is 30%–40% in Asia (14) and 10%–15% in Europe among the Caucasian population (15). Targeted therapies including gefitinib, erlotinib, and afatinib have been proved to effectively treat non-small cell lung cancer (NSCLC) with specific gene mutations. Patients with lung adenocarcinoma are the main beneficiaries of targeted therapy (16). It has been widely demonstrated that the gene mutation status of NSCLC is closely related to gender, age, ethnicity, pathological type, stage of cancer, smoking, and other clinicopathological factors. A higher gene mutation rate was found in non-smoking young Asian female patients with lung adenocarcinoma, especially EGFR mutation, among which mutations in exon E19 and E21 represented the vast majority (87%) of all EGFR mutations in patients with lung cancer (17–21). The understanding of the genetic mutation status of different populations and clinicopathological characteristics is of great significance for rationalizing drug usage and adjusting the therapeutic regimen appropriately. The existing literature on gene mutation of single lung cancer is exhaustive, but there is insufficient research on clinicopathological and demographic characteristics related to gene mutation of multiple primary lung cancer using a comprehensive large sample study.

In our study, we mainly analyzed the relationship between gene mutation and demographic characteristics as well as clinicopathological features of multiple primary lung cancer (with solid or subsolid nodules ≤ 3 cm), aiming to acquire a comprehensive understanding of genetic mutation characteristics in multiple primary lung cancer to guide targeted therapy in clinical practice.

## 2 Materials and Methods

### 2.1 Case selection

We enrolled all patients who underwent surgery for multiple primary lung cancers between June 2020 and April 2022 at the Chongqing University Cancer Hospital (Chongqing, China), in this study. We adopted the prognostic criterion of multiple primary lung cancers proposed by the American College of Chest Physicians (ACCP) (22, 23) and Girard et al. (24) (**Figure S1**).

All excised nodules were subjected to gene analysis. All of the patients underwent contrast-enhanced thoracic CT scans before surgery. Other routine preoperative examinations included chest radiograph, cardiopulmonary function tests, abdominal and adrenal gland ultrasonography or CT, brain magnetic resonance imaging (MRI) or CT, and a bone scan. Patients with suspected metastasis prior to surgery were excluded. Written informed patient consent was obtained for tissue analysis before surgery, and the protocol of this retrospective study was approved by the Institutional Review Board of Chongqing University Cancer Hospital.

### 2.2 Demographic and clinical variables

We collected the following demographic and clinical variables of patients: age, gender, tumor history, smoking history, drinking history, number of nodules, N staging, histology, histological subtype, gene mutation, local invasion of tumor, and tumor location and size measured by CT.

### 2.3 Samples and mutation detection

Formalin-fixed paraffin-embedded (FFPE) tumor tissues, or fine‐needle aspiration and/or core needle biopsies, were used to detect mutations in at least one of the following genes, EGFR, ALK, ROS1, KRAS, BRAF, RET, MET, HER2, NRAS, and PIK3CA. Genomic DNA and total RNA were extracted from FFPE samples using the AmoyDx FFPE DNA/RNA extraction kit (Amoy Diagnostics, Xiamen, China) following the manufacturer’s protocols. For other types of samples, an AmoyDx Tissue DNA/RNA extraction kit (Amoy Diagnostics) was used. An Amplification Refractory Mutation System Polymerase Chain Reaction (ARMS-PCR) and a Mutation Detection Kit (Amoy Diagnostics) were used to detect the mutations in driver genes (n = 2416).

### 2.4 DNA and RNA extraction

DNA and RNA were extracted from paraffin specimens strictly in accordance with the instructions in the AmoyDx FFPE DNA/RNA Kit (AmoyDx Inc, Xiamen, China), and DNA and RNA which were extracted from fresh cytological specimens strictly in accordance with the instructions in the AmoyDx Tissue DNA/RNA Kit (AmoyDx Inc, Xiamen, China) were checked for concentration and purity using a spectrophotometer (DeNovix, USA). The extracted DNA was stored in the refrigerator at -20°C, while the RNA was stored in the refrigerator at -70 °C for future use. OD260/OD280 was within 1.8∼2.0, the recommended concentration of the DNA from fresh specimens was 0.4-1 ng/μl.

### 2.5 ARMS for the gene mutation detection

We used AmoyDx Multi-Gene Mutations Detection kit and AmoyDx MET Mutation Detection Kit (AmoyDx Inc, Xiamen, China) to detect genes of EGFR, KRAS, BRAF, NRAS, HER2, and PIK3CA mutations, EML4-ALK, ROS1, and RET fusions, and used ABI 7500 Fluorescence quantitative PCR to detect MET Exon 14 Skipping mutation in accordance with the instructions provided.

### 2.6 DNA/RNA-based NGS

10 ng DNA was used as a template to construct the amplicon libraries using Thermo Fisher scientific ION Proton(DA8600) platform. The custom-designed panel encompassed 60 cancer-related genes, including AKT1, ALK, BRAF, CTNNB1, DDR2, EGFR, ERBB2, ERBB4, FGFR1, FGFR2, FGFR3, GNAQ, IDH2, JAK1, JAK2, KRAS, MAP2K1, MET, MEK1, NOTCH1, NRAS, PIK3CA, PTEN, PIK3CA, SMAD4, STK11, and TP53 etc.

RNA-based NGS was conducted using a custom-designed panel which included probes spanning the MET exon 13-15 junction. Also, the fusions of ALK, RET, ROS1, and NTRK1 were detected. The libraries were prepared using multiple PCR capture method and Ion Torrent high-throughput sequencing. Then, the amplicon libraries were sequenced using an Ion Torrent Systems Proton system and a PI chip with barcoding performed using an Ion Xpress Barcode Adapter 1-96 Kit (Thermo Fisher Scientific, MA, USA).

### 2.7 NGS data analysis

Torrent Suite Software (version 5.0) was used to perform signal processing, base calling, quality score assignment, and adapter trimming after sequencing reaction. The mutant allele frequency ≥ 1%, fusion mutants with ≥ 1000× coverage, and NDF ≥ -2.8 were accepted.

### 2.8 Statistical analysis

All statistical analyses were performed using the rms package of R language (version 4.2.1). The significance of the correlation between clinicopathological parameters and gene mutation status was analyzed using the Pearson’s chi-square test for categoric variables, and Fisher’s exact test. All tests were 2-sided and differences were considered significant at *P* < 0.05.

## 3 Results

### 3.1 Case selection and clinical parameters of patients

The results of the analysis of 265 tumors from 111 patients with multiple primary lung cancers diagnosed between June 2020 and April 2022 were retrieved (**Figure S1**). Cases with obvious intrapulmonary metastasis, as determined based on both histological and clinical assessments, were excluded. The mean age of the patients was 53.07 years (range 27–77 years). The number of female patients (n = 86; 77.5%) was much higher than that of male patients (n = 25; 22.5%). Ninety-eight patients (88.3%) did not have a history of smoking and 95 patients (85.6%) did not have a history of drinking. Only 6 patients (5.4%) had a family history of lung cancer. The median size of the largest nodule for each patient was 1 cm (range 0.2–3 cm). The number of nodules was 2 in 86 patients (77.5%) and the most nodules we found in one patient was 9. Multiple pulmonary nodules in most patients were located in the different lobes of the unilateral side (n = 48; 43.2%), followed by those located in the same lobe of the unilateral side (n = 43; 38.7%). Minimally invasive adenocarcinoma (n = 55; 49.5%) was the common histology for the main nodules, and were the largest nodules in one patient. Lymph node metastasis was identified in 4 patients (3.6%). The main nodules in 91 patients (82%) were detected to be mutated. We identified both perineural and vascular invasion in only 1 patient, however, 12 nodules from 12 patients (10.8%) had visceral pleural invasion. The median follow-up time was 1 month and the longest follow-up time was about 7 years. Two patients had metachronous multiple primary lung tumors, and the interval between the tumor appearances was 13 months and 27 months, respectively. Other demographic characteristics are summarized in **Table 1**.

**Table 1:** Demographic characteristics of patients.

Parameters	Number of patients (%)
Age (years; mean (SD))	
	53.07 ± 10.18
Sex	
Female	86 (77.5)
Male	25 (22.5)
History of drinking (%)	
No	95 (85.6)
Yes	16 (14.4)
History of smoking (%)	
No	98 (88.3)
Yes	13 (11.7)
Family history of lung cancer (%)	
No	105 (94.6)
Yes	6 (5.4)
Largest tumor size (cm;median [IQR])	
	1.00 [0.70, 1.85]
Number of nodules (%)	
2	86 (77.5)
3	15 (13.5)
4	5 (4.5)
5	4 (3.6)
9	1 (0.9)
Nodule location (%)	
Unilateral side, different lobe	48 (43.2)
Unilateral side, same lobe	43 (38.7)
Bilateral side	20 (18.0)
Histology (%)	
Carcinoma in situ	9 (8.1)
Minimally invasive adenocarcinoma	55 (49.5)
Invasive adenocarcinoma	47 (42.3)
Histological subtype (%)	
Carcinoma in situ	9 (8.1)
Minimally invasive adenocarcinoma	55 (49.5)
Lepidic predominant adenocarcinoma	4 (3.6)
Acinar adenocacinoma	37 (33.3)
Papillary adenocarcinoma	2 (1.8)
Papillary and acinar adenocarcinoma	2 (1.8)
Not otherwisespecified adenocarcinoma	2 (1.8)
N staging (%)	
N0	107 (96.4)
N1	4 (3.6)
Gene mutation (%)	
No	20 (18.0)
Yes	91 (82.0)
Perineural invasion (%)	
No	110 (99.1)
Yes	1 (0.9)
Vascular invasion (%)	
No	110 (99.1)
Yes	1 (0.9)
Visceral pleural invasion (%)	
No	99 (89.2)
Yes	12 (10.8)
Follow-up time (median [IQR])	
	1.00 [0.32, 4.50]
Synchronous vs. Metachronous (%)	
Metachronous	2 (1.8)
Synchronous	109 (98.2)

### 3.2 Clinicopathologic features of patients with different gene mutation status

A summary of the analysis of the gene mutation status and clinicopathological parameters is given in **Table 2**. The incidence of gene wildtype in ≥ 65 years old group was higher than those having at least one mutated-gene tumor (*P* = 0.024) The smoking ratio in patients with all mutated-gene nodules (n = 9, 19.6%) was higher than those with partial mutated-gene nodules (n = 2, 4.2%) (*P* = 0.002). However, there were no significant differences in the smoking ratio among patients with or without gene mutations (*P* = 1.000). The ratio of ≥ 3 nodules among patients having partial mutated-gene nodules was higher than those having all mutated-gene nodules (*P* < 0.001). The main nodules of patients with wildtype gene were more likely to be detected in the same lobe of the unilateral side, while those of patients with partial gene mutation and all gene mutation were more likely to be detected in different lobes of the unilateral side (*P* = 0.013). For the rest, the clinicopathologic features were not significantly different among patients having all wildtype gene tumors, those having all mutated-gene tumors, and those having partial mutated-gene tumors.

**Table 2:** Comparison of demographic parameters and clinicopathologic feature between patients with Gene-wildtype tumors and Gene-mutant tumors.

	Gene Mutant				
Parameters	Gene Mutant/Mutant (n = 46)	Gene Mutant/Wildtype (n = 48)	p-Value	Gene Wildtype (n = 17)	p-Value
Age (year, %)					
<65	41 (89.1)	46 (95.8)		12 (70.6)	
>=65	5 (10.9)	2 (4.2)	0.398	5 (29.4)	0.024
Sex (%)					
Female	34 (73.9)	40 (83.3)		12 (70.6)	
Male	12 (26.1)	8 (16.7)	0.265	5 (29.4)	0.672
History of drinking (%)					
No	38 (82.6)	42 (87.5)		15 (88.2)	
Yes	8 (17.4)	6 (12.5)	0.506	2 (11.8)	1.000
History of smoking (%)					
No	37 (80.4)	46 (95.8)		15 (88.2)	
Yes	9 (19.6)	2 (4.2)	0.002	2 (11.8)	1.000
Family history of lung cancer (%)					
No	44 (95.7)	45 (93.8)		16 (94.1)	
Yes	2 (4.3)	3 (6.2)	1.000	1 (5.9)	1.000
Largest tumor size (cm, %)					
<=1	18 (39.1)	30 (62.5)		10 (58.8)	
>1 ~<=2	15 (32.6)	12 (25.0)		3 (17.6)	
>2 ~<=3	13 (28.3)	6 (12.5)	0.053	4 (23.5)	0.690
Number of nodules (%)					
2	42 (91.3)	30 (62.5)		14 (82.4)	
>=3	4 (8.7)	18 (37.5)	<0.001	3 (17.6)	0.836
Nodule location (%)					
Unilateral side, same lobe	17 (37.0)	14 (29.2)		12 (70.6)	
Unilateral side, different lobe	22 (47.8)	23 (47.9)		3 (17.6)	
Bilateral side	7 (15.2)	11 (22.9)	0.561	2 (11.8)	0.013
Histology (%)					
Carcinoma in situ	1 (2.2)	7 (14.6)		1 (5.9)	
Minimally invasive adenocarcinoma	22 (47.8)	22 (45.8)		11 (64.7)	
Invasive adenocarcinoma	23 (50.0)	19 (39.6)	0.09	5 (29.4)	0.397
N staging (%)					
N0	43 (93.5)	47 (97.9)		17 (100.0)	
N1	3 (6.5)	1 (2.1)	0.579	0 (0.0)	1.000
Perineural invasion (%)					
No	45 (97.8)	48 (100.0)		17 (100.0)	
Yes	1 (2.2)	0 (0.0)	0.489	0 (0.0)	1.000
Vascular invasion (%)					
No	45 (97.8)	48 (100.0)		17 (100.0)	
Yes	1 (2.2)	0 (0.0)	0.489	0 (0.0)	1.000
Visceral pleural invasion (%)					
No	41 (89.1)	45 (93.8)		13 (76.5)	
Yes	5 (10.9)	3 (6.2)	0.665	4 (23.5)	0.158
Synchronous vs. Metachronous (%)					
Synchronous	46 (100.0)	46 (95.8)		17 (100.0)	
Metachronous	0 (0.0)	2 (4.2)	0.495	0 (0.0)	1.000

### 3.3 Concordance of gene mutation status

**Figure 1:**
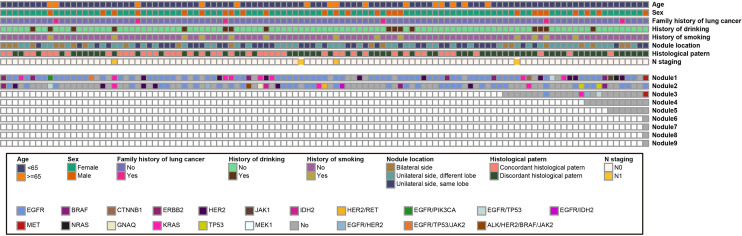
Schematic demonstration of the clinicopathological parameters and gene mutationColumns represent different patients and rows represent different clinical or pathological information. The different colors are annotated with clinical and pathological information at the bottom of the chart.

In this study, 63 patients (56.8%) had different gene mutation statuses (15 patients had different mutations, and 48 patients had at least one wildtype gene tumor), 31 patients (27.9%) had the same gene mutations, and 17 patients (15.3%) had only wildtype gene tumors. The clinicopathological data and gene mutation status are shown in **Figure 1**. The most frequently detected gene mutation among the patients was EGFR (detected in 100 nodules of 70 patients), followed by HER2 (detected in 15 nodules of 14 patients). The results of comparative analysis of clinicopathological parameters between patients with the same mutation and those with different mutations are summarized in **Table 3**. The size of pulmonary nodules between different gene mutation groups (n = 38, 57.6%) was more likely ≤ 1 cm, while that among the same gene mutation group (n = 11, 39.3%) was > 2–3 cm (*P* =0.015). Additionally, the number of pulmonary nodules of > 3 in different gene mutation groups (n = 20, 31.7%) was significantly lower than that in the same gene mutation group (n = 28, 92.9%) (*P* = 0.015). Other clinicopathological parameters were not significantly different.

**Table 3:** Comparison of demographic parameters and clinicopathologic feature between patients with same gene mutation and those with different gene mutations.

Parameters	Different (n = 63)	Same (n = 31)	p-Value
Age (year, %)			
<65	60 (95.2)	26 (85.7)	
>=65	3 (4.8)	5 (14.3)	0.251
Sex (%)			
Female	48 (76.2)	25 (82.1)	
Male	15 (23.8)	6 (17.9)	0.527
History of drinking (%)			
No	52 (82.5)	27 (89.3)	
Yes	11 (17.5)	4 (10.7)	0.611
History of smoking (%)			
No	56 (88.9)	26 (85.7)	
Yes	7 (11.1)	5 (14.3)	0.936
Family history of lung cancer (%)			
No	60 (95.2)	29 (93.5)	
Yes	3 (4.8)	2 (6.5)	1
Largest tumor size (cm, %)			
<=1	35 (55.6)	11 (35.7)	
>1~<=2	20 (31.7)	8 (25.0)	
>2 ~<=3	8 (12.7)	12 (39.3)	0.015
Number of nodules (%)			
2	43 (68.3)	3 (7.1)	
>=3	20 (31.7)	28 (92.9)	<0.001
Nodule location (%)			
Unilateral side, same lobe	19 (30.2)	11 (35.7)	
Unilateral side, different lobe	31 (49.2)	14 (46.4)	
Bilateral side	13 (20.6)	5 (17.9)	0.863
Histology (%)			
Carcinoma in situ	6 (9.5)	2 (3.6)	
Minimally invasive adenocarcinoma	31 (49.2)	12 (39.3)	
Invasive adenocarcinoma	26 (41.3)	17 (57.1)	0.347
Histological subtype (%)			
Carcinoma in situ	6 (9.5)	2 (3.6)	
Minimally invasive adenocarcinoma	31 (49.2)	12 (39.3)	
Acinar adenocacinoma	22 (34.9)	12 (39.3)	
Lepidic predominant adenocarcinoma	2 (3.2)	2 (7.1)	
Papillary adenocarcinoma	1 (1.6)	1 (3.6)	
Papillary and acinar adenocarcinoma	1 (1.6)	1 (3.6)	
Not otherwisespecified adenocarcinoma	0(0.0)	1 (3.6)	0.455
N staging (%)			
N0	62 (98.4)	28 (90.3)	
N1	1 (1.6)	3 (9.7)	0.159
Perineural invasion (%)			
No	62 (98.4)	31 (100.0)	
Yes	1 (1.6)	0 (0.0)	1
Vascular invasion (%)			
No	62 (98.4)	31 (100.0)	
Yes	1 (1.6)	0 (0.0)	0.308
Visceral pleural invasion (%)			
No	59 (93.7)	27 (85.7)	
Yes	4 (6.3)	4 (14.3)	0.405
Synchronous vs. Metachronous (%)			
Metachronous	2 (3.2)	0(0.0)	
Synchronous	61 (96.8)	31 (100.0)	1

### 3.4 Clinicopathological features of nodules

As seen in **Table 4**, we found that 109 nodules (41.1%) were wildtype in all the 265 nodules from the 111 patients, and in the remaining 156 nodules (58.9%), the ones with EGFR mutation (n = 99, 37.4%) were more common than those with other type gene mutation (n = 57, 21.5%). Multiple nodules were most commonly found in the upper lobe of the right lung (n = 105, 39.6%), followed by the upper lobe of the left lung (n = 59, 22.3%), while they were the least commonly detected in the right middle lobe of the lung (n = 25, 9.4%). In histological analysis, the most common histology of nodules was the minimally invasive adenocarcinoma (n = 143, 54.0%), and squamous cell carcinoma (n = 1, 0.4%) was very rare. Moreover, invasive adenocarcinoma could be further classified into different histological subtypes, namely, lepidic adenocarcinoma, acinar adenocarcinoma, and papillary adenocarcinoma, with acinar adenocarcinoma (n = 65, 24.5%) being the most dominant histological subtype of invasive adenocarcinoma. Among the 265 nodules, 198 nodules (74.7%) were ≤ 1 cm and 25 nodules (9.4%) were around 2 cm ~ 3 cm. In addition, there was only 1 nodule (0.4%) that appeared in both vascular and nerve invasion, but 12 nodules (4.5%) appeared with visceral pleural invasion.

**Table 4:** Clinicopathologic feature of nodules.

Parameters	Numbers (n = 265)
Tumor size (%)	
<=1	198 (74.7)
>1~<=2	42 (15.8)
>2 ~<=3	25 (9.4)
Gene (%)	
EGFR	99 (37.4)
Else	57 (21.5)
No	109 (41.1)
Nodule location (%)	
Left lower lobe	31 (11.7)
Left upper lobe	59 (22.3)
Right lower lobe	45 (17.0)
Right middle lobe	25 (9.4)
Right upper lobe	105 (39.6)
Histology (%)	
Carcinoma in situ	41 (15.5)
Minimally invasive adenocarcinoma	143 (54.0)
Invasive adenocarcinoma	80 (30.2)
Squamous carcinoma	1 (0.4)
Histological subtype (%)	
Carcinoma in situ	41 (15.5)
Acinar adenocacinoma	65 (24.5)
Lepidic predominant adenocarcinoma	6 (2.3)
Minimally invasive adenocarcinoma	143 (54.0)
Papillary adenocarcinoma	3 (1.1)
Papillary and acinar adenocarcinoma	3 (1.1)
Not otherwisespecified adenocarcinoma	3 (1.1)
Well-differentiated squamous cell carcinoma	1 (0.4)
Perineural invasion (%)	
No	264 (99.6)
Yes	1 (0.4)
Vascular invasion (%)	
No	264 (99.6)
Yes	1 (0.4)
Visceral pleural invasion (%)	
No	253 (95.5)
Yes	12 (4.5)

### 3.5 Clinicopathologic features of nodules with different gene mutation status

In this study, we further evaluated the clinicopathological features of nodules according to the gene mutation status (**Table 5**). In the analysis of tumor size, we found that the percentage of nodules ≤ 1 cm in the other gene mutation (n = 45, 78.9%) or the wildtype gene group (n = 95, 87.2%) was higher than in the EGFR mutation group (n = 58, 58.6%) (*P* < 0.001). On the contrary, the percentage of nodules > 2 cm but ≤ 3 cm in the other gene mutation (n = 0, 0.0%) or the wildtype gene group (n = 7, 6.4%) was smaller than in the EGFR mutation group (n = 14, 14.1%) (*P* < 0.001). Nodules with EGFR mutation were more likely to be present in the left upper lobe of the lung (n = 31, 31.3%), while those with other type mutation (n = 23, 40.3%) or wildtype gene (n = 52, 47.7%) were more likely to be present in the right upper lobe of the lung (*P* = 0.007). In the histological analysis, invasive adenocarcinoma was the most common type detected in nodules with the EGFR mutation (n = 54, 54.5%), while minimally invasive adenocarcinoma was more likely to be detected in those with other type gene mutation (n = 37, 64.9%) or wildtype gene (n = 66, 60.6%). Furthermore, we also found that the frequency of carcinoma *in situ* among the nodules with other type gene mutation (n = 8, 14.1%) or wildtype gene (n = 28, 25.7%) was higher than those with EGFR mutation (n = 5, 5.1%) (*P* < 0.001). Additionally, we found that acinar adenocarcinoma was the main histological subtype of the invasive adenocarcinoma irrespective of the gene mutation status of the nodules. The only nodule with both perineural and vascular invasion was EGFR-mutant (*P* = 0.049). There was no obvious difference between perineural, vascular, and visceral pleural invasion among the nodules of different gene mutations.

**Table 5:** Clinicopathologic feature of nodules based on different gene mutation and wildtype-gene.

Parameters	EGFR mutation(n = 99)	Othertype gene mutation (n = 57)	Gene-wildtype (n = 109)	p-value
Tumor size (%)				
<=1	58 (58.6)	45 (78.9)	95 (87.2)	
>1~<=2	27 (27.3)	8 (14.0)	7 (6.4)	
>2 ~<=3	14 (14.1)	0(0.0)	7 (6.4)	<0.001
Nodule location				
Left lower lobe	10 (10.1)	12 (21.1)	9 (8.3)	
Left upper lobe	31 (31.3)	5 (8.8)	23 (21.1)	
Right lower lobe	16 (16.2)	10 (17.5)	19 (17.4)	
Right middle lobe	12 (12.1)	7 (12.3)	6 (5.5)	
Right upper lobe	30 (30.3)	23 (40.3)	52 (47.7)	0.007
Histology (%)				
Carcinoma in situ	5 (5.1)	8 (14.1)	28 (25.7)	
Minimally invasive adenocarcinoma	40 (40.4)	37 (64.9)	66 (60.6)	
Invasive adenocarcinoma	54 (54.5)	12 (21.1)	14 (12.8)	
Squamous carcinoma	0(0.0)	0(0.0)	1 (0.9)	<0.001
Histological subtype (%)				
Carcinoma in situ	5 (5.1)	8 (14.0)	28 (25.7)	
Minimally invasive adenocarcinoma	40 (40.4)	37 (64.9)	66 (60.6)	
Acinar adenocacinoma	46 (46.5)	10 (17.5)	9 (8.3)	
Lepidic predominant adenocarcinoma	3 (3.0)	0(0.0)	3 (2.8)	
Papillary adenocarcinoma	2 (2.0)	1 (1.8)	0(0.0)	
Papillary and acinar adenocarcinoma	2 (2.0)	1 (1.8)	0(0.0)	
Not otherwisespecified adenocarcinoma	1 (1.0)	0(0.0)	2 (1.8)	
Well-differentiated squamous cell carcinoma	0(0.0)	0(0.0)	1 (0.9)	<0.001
Perineural invasion (%)				
No	98 (99.0)	57 (100.0)	109 (100.0)	
Yes	1 (1.0)	0 (0.0)	0 (0.0)	0.589
Vascular invasion (%)				
No	98 (99.0)	57 (100.0)	109 (100.0)	
Yes	1 (1.0)	0 (0.0)	0 (0.0)	0.589
Visceral pleural invasion (%)				
No	94 (94.9)	55 (96.5)	104 (95.4)	
Yes	5 (5.1)	2 (3.5)	5 (4.6)	1

### 3.6 Clinicopathologic feature of nodules with different EGFR mutation status

EGFR gene mutation is the most common and important type of gene mutation in lung adenocarcinoma, we conducted subgroup analysis on 99 EGFR gene mutation nodules. We found that the proportion of microinvasive adenocarcinoma in lung nodules with 19/21 gene mutation was relatively high (n=11,68.8%), while the proportion of invasive adenocarcinoma was relatively low (n=4,5%); on the contrary, the proportion of infiltrating adenocarcinoma in lung nodules with 18/20 gene mutation is relatively high (n=50, 60.2%), while the proportion of micro-invasive adenocarcinoma is relatively low (n=29, 34.9%).There was no obvious difference between tumor size, nodule location, histological subtype, perineural, vascular, and visceral pleural invasion among the nodules of different EGFR mutations.

## 4 Discussion

In this study, we found that the main nodule in all cases was adenocarcinoma or carcinoma in situ, consisting of the pathology of malignant solitary pulmonary ground-glass nodules (25). Our findings showed the age of onset of multiple primary lung cancer as 53.07 ± 10.18 years, which is similar to previous reports (21). Among the 111 patients, there were 86 females, accounting for 77.5%. Previous studies also suggested that adenocarcinoma is more common in women (26). The difference we found was that only 13 (11.7%) of the patients with multiple primary lung cancers smoked, which was much lower than that of 88.2% (27) in patients with lung adenocarcinoma in European and American countries, and Asian patients carrying genetic mutations with lung cancer were predominantly female and had adenocarcinoma. In 6 Asian trials that consisted of patients with only EGFR mutations, 27.9% of the 1,416 patients (that is, 395/1,416 cases) were former or current smokers (28–35). Thus, these findings indicate the importance of screening programs in the non-smoker subgroup (36). Further analysis showed that the 13 smoking patients were all male, that is, about half (13/25 cases, or 52%) of the male patients with multiple primary early-stage lung cancer had a history of smoking. However, it remains to be further confirmed whether smoking is a risk factor for multiple primary early-stage lung cancer in males. Commonly, smoking and exposure to kitchen fumes are high-risk factors for lung cancer in women. Up to 90% of non-smoking women in East Asia carry driver genes, and approximately 70% of patients have access to targeted drug therapy (37). All the enrolled female patients declared no smoking history, with most of the main nodules (56/86 cases, or 65%) associated with genetic mutations. Hence, we speculate that the driver genes may be closely related to the occurrence of multiple primary lung cancer in women. The main lesion in 47 patients (42.3%) was invasive carcinoma, with the rest having microinvasive adenocarcinoma or primary carcinoma, with only 4 cases of N1 (3.6%), indicating that the early stage of multiple primary lung cancers may be relatively inert and slow in growth. In this study, the longest followed-up was Subject 51, a 63-year-old female, with a follow-up time of up to 7 years. There were 3 nodules, which were micro-invasive adenocarcinoma *in situ* and invasive adenocarcinoma, in different lobes of the right lung. It is worth mentioning that only 1 patient (0.9%) had concurrent nerve and vascular invasion, but 12 patients (10.8%) showed visceral pleural invasion. This may be mainly because of the peripheral type distribution of primary lung cancer and some nodules appear easily in the subpleural. Therefore, we suggest that multiple pulmonary nodules near the pleura should be treated actively.

The history of smoking in patients acquiring mutant/mutant phenotype (9 cases, or 19.6%) was significantly higher than patients acquiring mutant/wildtype (2 cases, or 4.2%), contradictory to the fact that lung cancer gene mutations were more common in non-smoking patients (38–41). Among patients who had ≥ 3 pulmonary nodules, there were 18 with mutant/wildtype genotypes (37.5%), which was significantly higher than 4 cases of patients having mutant/mutant genotypes (8.7%). This may be related to the randomness of gene mutations in pulmonary nodules. That is, the more the multiple pulmonary nodules, the higher the probability of acquiring genetic mutants and wildtype. There were 5 (29.4%) patients over 65 years old who appeared in WT, and only 7 (7.6%) patients with at least one gene mutation were over 65 years old, indicating a lower probability of gene mutation in the elderly than that in the middle-aged in multiple primary lung cancer. Pulmonary nodules were more likely to be distributed in different lobes of the same lung in patients with gene mutation (45/94 cases, or 47.8%), while WT was more likely to be distributed in the same lobe of the same lung (12 cases, or 70.6%). As per the remaining results, patients with multiple lung cancers, with at least one gene mutation, and those with multiple lung cancers with all EGFR-wildtype tumors did not exhibit different demographic characteristics.

Among the 111 patients, 94 patients acquired genetic mutations, with more than half of the cases (63/94 cases, or 67%) appearing to be inconsistent genetic mutations. Therefore, subjecting only one cancerous lesion to gene analysis cannot conclusively determine the gene mutation status in the remaining tumors. In patients with multiple lung cancers who underwent surgery for major lesions harboring EGFR mutations, postoperative EGFR-TKI treatment for unresected ground glass opacity (GGO) lesions decreased the size of residual GGOs, especially in patients with large or residual GGOs or advanced stage tumors. However, the size of residual GGOs in most patients remained unchanged (42). In our study, only 31% of patients with gene-mutant tumors had another same mutated gene tumor. These findings suggest the importance of determining the mutation status of each residual lesion when considering TKI treatment for patients with multiple lung cancers.

In patients with the same gene mutation, the largest proportion of main nodules was > 2 ≤ 3 cm (12 cases, or 39.3%), and the number of nodules was ≥ 3 (28 cases, or 92.9%). While in patients with different gene mutations, the largest proportion of main nodules was ≤ 1 cm (35 cases, or 55.6%), and the number of nodules was mainly 2 (43 cases, or 68.3%). It is evident that patients with the same gene mutation tend to have more nodules than those with different gene mutations, indicating a worse prognosis.

In the 256 nodules of all patients, EGFR mutation was frequently detected in tumors located in the upper portion of the lung (79/113 cases, or 69.9%) and adenocarcinoma was common (111/113 cases, or 98.2%), especially in tumors with acinar-predominant pattern (85/113 cases, or 76.6%), which is similar to other studies (41, 43, 44). EGFR mutation nodules were more likely to occur in the right upper lobe (30/99 cases, or 30.3%) and left upper lobe (31/99 cases, or 31.3%). While nodules with gene mutation of other types (23/57 cases, or 40.3%) and WT (52/109 cases, or 47.7%) nodules were more prevalent in the right upper lobe than in the left upper lobe.

Nodules with EGFR mutation > 2 cm ~ ≤ 3cm (14/99 cases, or 14.1%) accounted for the largest proportion when compared to those with other gene mutations (0/57 cases, or 0.0%) and wildtype (7/109 cases, or 6.4%), with invasive adenocarcinoma (54/99 cases, or 54.5%) as the main type clinically, while other type gene mutation (37/57 cases, or 64.9%) and wildtype (66/109 cases, or 60.6%) were mainly characterized by earlier microinvasive adenocarcinoma. This finding is consistent with the hypothesis that EGFR mutations are acquired during the development of invasive adenocarcinoma from pre-invasive lesions, such as atypical adenomatous hyperplasia (AAH), and that they accelerate the progression of tumors rather than (45) initiate tumorigenesis. Additionally, EGFR mutations were acquired independently in precancerous lesions with multifocal presentations (46), even though the same patient exhibited different EGFR statuses in individual tumors. EGFR mutations mainly include 18/19/20/21 gene mutations, of which 19 and 21 gene mutations are the most common and the targeted drug treatment effect is clearer, while 18/20 gene mutations are rare and the targeted drug treatment effect is relatively uncertain. Therefore, we conducted a subgroup analysis of clinical relevance of 99 EGFR gene mutations in pulmonary nodules according to the gene mutation sites. We found the proportion of micro-invasive adenocarcinoma was higher in pulmonary nodules with 19/21 gene mutation than that with 18/20 gene mutation, which suggested the degree of malignancy of pulmonary nodules was relatively weak. In addition, pulmonary nodules with 19/21 gene mutation are more sensitive to targeted drug treatment, which may mean the prognosis of such pulmonary nodules is better than that of pulmonary nodules with 18/20 gene mutation (**Table 6**).

**Table 6:** Clinicopathologic feature of nodules based on different EGFR mutation.

Parameters	19/21 gene mutation (n = 16)	18/20 gene mutation (n = 83)	p-Value
Tumor size (%)			
<=1	12 (75.0)	46 (55.4)	
>1~<=2	2 (12.5)	25 (30.1)	
>2 ~<=3	2 (12.5)	12 (14.5)	0.2951
Nodule location			
Left lower lobe	2 (12.5)	8 (9.6)	
Left upper lobe	6 (37.5)	25 (30.1)	
Right lower lobe	5 (31.2)	11 (13.3)	
Right middle lobe	2 (12.5)	10 (12.0)	
Right upper lobe	1 (6.2)	29 (34.9)	0.154
Histology (%)			
Carcinoma in situ	1 (6.2)	4 (4.8)	
Minimally invasive adenocarcinoma	11 (68.8)	29 (34.9)	
Invasive adenocarcinoma	4 (25.0)	50 (60.2)	0.03169
Histological subtype (%)			
Carcinoma in situ	1 (6.2)	4 (4.8)	
Minimally invasive adenocarcinoma	11 (68.8)	29 (34.9)	
Acinar adenocacinoma	4 (25.0)	42 (50.6)	
Lepidic predominant adenocarcinoma	0 (0.0)	3 (3.6)	
Papillary adenocarcinoma	0 (0.0)	2 (2.4)	
Papillary and acinar adenocarcinoma	0 (0.0)	2 (2.4)	
Not otherwisespecified adenocarcinoma	0 (0.0)	1 (1.2)	0.2954
Perineural invasion (%)			
No	16 (100.0)	82 (98.8)	
Yes	0 (0.0)	1(1.2)	1
Vascular invasion (%)			
No	16 (100.0)	82 (98.8)	
Yes	0 (0.0)	1 (1.2)	1
Visceral pleural invasion (%)			
No	15 (93.8)	79 (95.2)	
Yes	1 (6.2)	4 (4.8)	1

Although our research has a large research queue in the field, there are still some limitations. Firstly, participant enrollment. We included only patients with multiple primary early lung cancers, most of whom had ground-glass pulmonary nodules that generally indicate early lung cancer on imaging, to avoid the dilution of results due to the inclusion of intrapulmonary metastases. At the same time, as the most common clinical manifestations of multiple pulmonary nodules on imaging, ground-glass is representative. Secondly, although we included patients in strict accordance with the diagnostic criteria of multiple primary lung cancer, we still could not guarantee the possibility of mixed metastatic cancer cases in the lung, so it might have a certain bias on the results. Thirdly, this research lacked a control group as this was an observational study. Forthly, the study did not involve prognosis follow-ups, due to which the influence of different gene mutations status on prognosis remains unclear. Last. There may be possible confounding factors resulting from the study being restricted to a single center. These limitations must be addressed in future studies.

## 5 Conclusion

This study revealed the demographic and clinicopathological features of patients with early multiple primary lung cancer. We found that the gene mutation status between different nodules in patients with early multiple primary lung cancer was inconsistent in most cases. Therefore, the use of targeted therapy based on the genetic sequencing of only one nodule, is unreliable. We hope that this study can assist in enhancing the understanding of genetic mutation characteristics in multiple primary lung cancer and offer clinical guidance for physicians.

## Data availability statement

The original contributions presented in the study are included in the article/**Supplementary Material**. Further inquiries can be directed to the corresponding authors.

## Ethics statement

The study was conducted in accordance with the Declaration of Helsinki (as was revised in 2013). The study was approved by Ethics Committee of the Chongqing University Cancer Hospital. The patients/participants provided their written informed consent to participate in this study.

## Author contributions

Conception and design of the research: Y-QJ, FT, Z-QW. Acquisition of data: FT, Z-QW, JX. Analysis and interpretation of the data: BY, Y-ZW. Statistical analysis: FT, JW, JX. Obtaining financing: Y-QJ, Y-ZW, FT. Writing of the manuscript: FT, JX. Critical revision of the manuscript for intellectual content: Y-QJ, FT. All authors read and approved the final draft. All authors contributed to the article and approved the submitted version.

## Supplementary material

Supplementary Figure 1 Workflow for patients’ selectionMultiple lung nodules with different pathological types are considered as multiple primary lung cancer; Multiple lung nodules with the same pathological type and located in the same lung lobe were considered as intrapulmonary metastatic carcinoma; Multiple lung nodules have the same pathological type and are located in different lobes. If there is no N2/N3 lymph node metastasis, they are considered as multiple primary lung cancer; Multiple lung nodules with the same pathological type and located in different lung lobes, if accompanied by N2/N3 lymph node metastasis, are considered as intrapulmonary metastatic cancer.
