# Supplementary figures and images for: Correlation between gene mutation status and clinicopathologic features in early multiple primary lung cancer
Source: Front Oncol. 2023 Apr 12;13:1110259. doi: 10.3389/fonc.2023.1110259 (PMC10130385; doi:10.3389/fonc.2023.1110259)

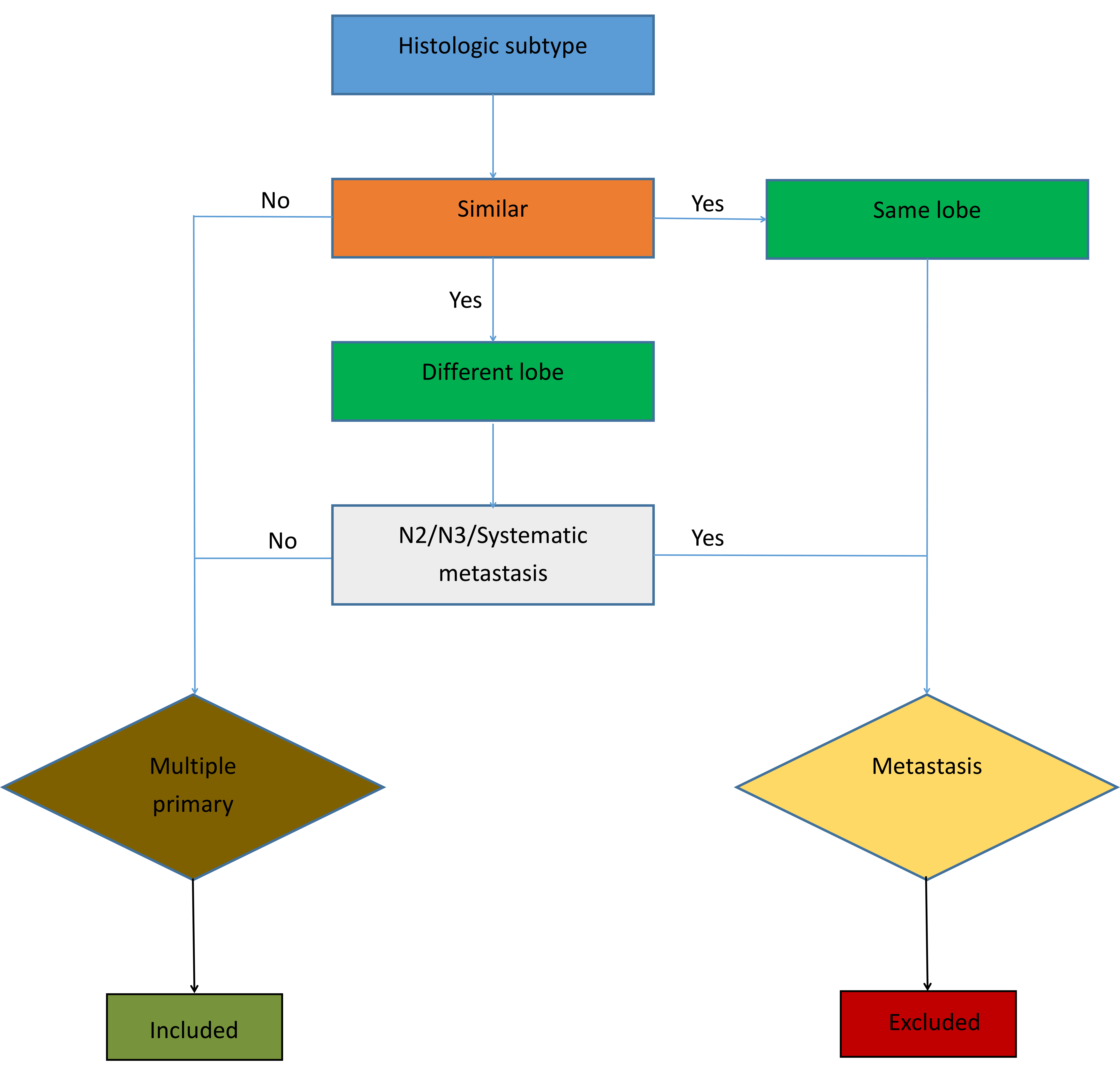

Supplement: Supplementary Figure 1 — Workflow for patients’ selectionMultiple lung nodules with different pathological types are considered as multiple primary lung cancer; Multiple lung nodules with the same pathological type and located in the same lung lobe were considered as intrapulmonary metastatic carcinoma; Multiple lung nodules have the same pathological type and are located in different lobes. If there is no N2/N3 lymph node metastasis, they are considered as multiple primary lung cancer; Multiple lung nodules with the same pathological type and located in different lung lobes, if accompanied by N2/N3 lymph node metastasis, are considered as intrapulmonary metastatic cancer. [file Image_1.jpeg]
